# FTO affects hippocampal function by regulation of BDNF processing

**DOI:** 10.1371/journal.pone.0211937

**Published:** 2019-02-07

**Authors:** André Spychala, Ulrich Rüther

**Affiliations:** Institute of Animal Developmental and Molecular Biology, Heinrich Heine University, Düsseldorf, Germany; Radboud University Medical Centre, NETHERLANDS

## Abstract

Initially, the function of the fat mass and obesity associated (Fto) gene seemed to be primarily the regulation of the body weight. Here we show that loss of Fto results in a hyperactivation of the hypothalamic-pituitary-adrenal (HPA) axis. In consequence, Fto^-/-^ mice display an anxiety-like behavior and impairments in working memory. Furthermore, differentiation of neurons is affected in the hippocampus. As a cause of these impairments we identified a processing defect of the neurotrophin BDNF which is most likely the result of a reduced expression of MMP-9. Therefore, we propose FTO as a possible new target to develop novel approaches for the treatment of diseases associated with hippocampal disorders. In parallel, we also would like to make the point that any anti-obesity therapy via blocking FTO function can have negative effects on the proper function of the hippocampus.

## Introduction

The Fto gene was first described as one of six deleted genes in the Fused-toes (Ft) mouse mutant [1]. Subsequently, we demonstrated that loss of Fto reduces adiposity and protects from diet-induced obesity and associated pathology such as insulin resistance [2, 3]. To date the FTO gene is discussed as a significant contributor to polygenetic obesity [4] and one promising key player in epigenetic obesity treatment. However, in the course of conducting large-scale genotyping studies, FTO seems to have a role in the central nervous system. It was shown that FTO is highly expressed in the brain and is essential for the correct development of the CNS in humans [5, 6]. In addition, SNPs within the FTO gene could be associated with neurological disorders such as Alzheimer’s and depression [7, 8]. Thus, these data provide strong evidence, that FTO is a key factor for CNS function and is implicated in CNS disorders. Recently it could be shown that FTO influences neurogenesis and that the loss of FTO could alter the brain-derived neurotrophic factor (BDNF) signaling pathway within the hippocampus [9].

The hippocampus as part of the limbic system plays an important role in the regulation of the stress response, cognitive functions and processing of external stimuli. Reduced neurogenesis and altered BDNF signaling in the hippocampus are mainly associated with the development of an array of adverse effects, such as mood alteration, induction of anxiety, cognitive dysfunction and hyperactivation of the hypothalamic-pituitary-adrenal (HPA) axis leading to impairments in the stress response [10–13]. BDNF is an important neurotrophic factor known to implement its action within the hippocampus by two different isoforms: mature BDNF (mBDNF) and precursor BDNF (proBDNF). Both isoforms preferentially bind specific receptors and exert distinct functions. While mBDNF activates NTRK2 receptor to promote survival, growth and differentiation, proBNDF binds to the NGFR receptor leading to neuronal cell death and synaptic withdrawal [14, 15].

Like most neurotrophins, BDNF is synthesized in the endoplasmic reticulum as a precursor, preproBDNF, and then processed to the precursor protein proBDNF. Further processing of proBDNF to mBDNF takes place by proteolytic cleavage, which can be realized both intracellularly and extracellularly. Intracellular processing in the trans-Golgi network can be performed by the endoprotease furin or in immature secretory vesicles by proprotein convertases [16, 17]. Extracellular processing is carried out by the proteolytic tPA/plasmin-cascade or the matrix metalloproteinases (MMP)-3, -7 and -9 [18, 19]. Various studies have shown that most of the processing from proBDNF to mBDNF occurs extracellularly in the nervous system and is dependent on the neuronal activity [19, 20].

Due to the contrasting effect of proBDNF and mBDNF on the function of neurons, the regulation of BDNF processing and the involvement in signaling pathways has become the focus of research in order to find possible new approaches for the treatment of neurological diseases [21].

Here we show that loss of FTO restricts various functions of the hippocampus. Thus, Fto deficiency leads to increased stress parameters such as corticosterone in the blood plasma, which implies hypersensitivity of the HPA axis. Furthermore, mice have deficits in working memory and an increased anxiety. As a cause a processing defect of neurotrophin BDNF could be found in the hippocampus of Fto^-/-^ mice. Together these results suggest that FTO may be a possible new target to find novel approaches for the treatment of neurological diseases via regulating BDNF processing.

## Results

The loss of FTO impairs HPA axis regulation and leads to higher stress hormone levels

**Fig 1:**
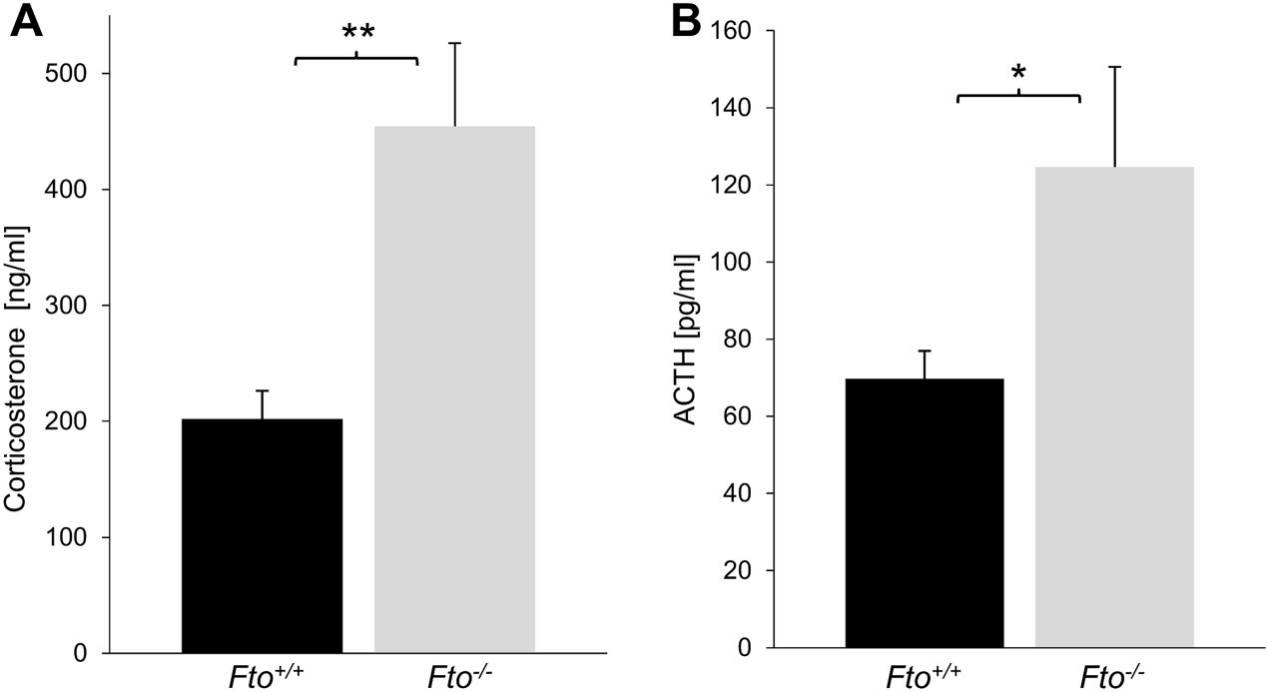
The loss of FTO leads increased levels of corticosterone and ACTH. (A-B) Quantification of plasma levels indicated that corticosterone (A) and ACTH (B) are significantly elevated in Fto^-/-^ mice, n = 16 Fto^+/+^ / 19 Fto^-/-^ (A); n = 13 Fto^+/+^ / 5 Fto^-/-^ (B). Data are presented as mean ± S.E.M., unpaired t-test, *P < 0.05; **P < 0.01.

In our mouse colony we observed that FTO deficient mice displayed an atypical behavior compared to control littermates such as jumping or tail beating indicating an increased fear or an elevated stress level. The HPA axis is an important neuroendocrine regulatory circuit, which is responsible for the transformation of stress-induced nerve impulses into hormonal signals such as corticosterone from the adrenal gland. Therefore, we analyzed the plasma levels of corticosterone under basal conditions in Fto^+/+^ and Fto^-/-^ mice. The quantification showed that the levels of corticosterone of Fto^-/-^ mice were significantly increased compared to Fto^+/+^ mice (Fig 1A). Next, we measured plasma levels of adrenocorticotropic hormone (ACTH), which mediates the release of corticosterone from the adrenal gland. As shown in Fig 1B, ACTH levels were significantly increased in Fto^-/-^ mice. These results demonstrate that Fto^-/-^ mice suffer from elevated levels of stress hormones. Furthermore, since we found a hyperactivation of the pituitary gland the data suggest that FTO has an important function in the regulation of the HPA axis.

### The loss of FTO leads to anxiety-like behavior and impairments in working memory

**Fig 2:**
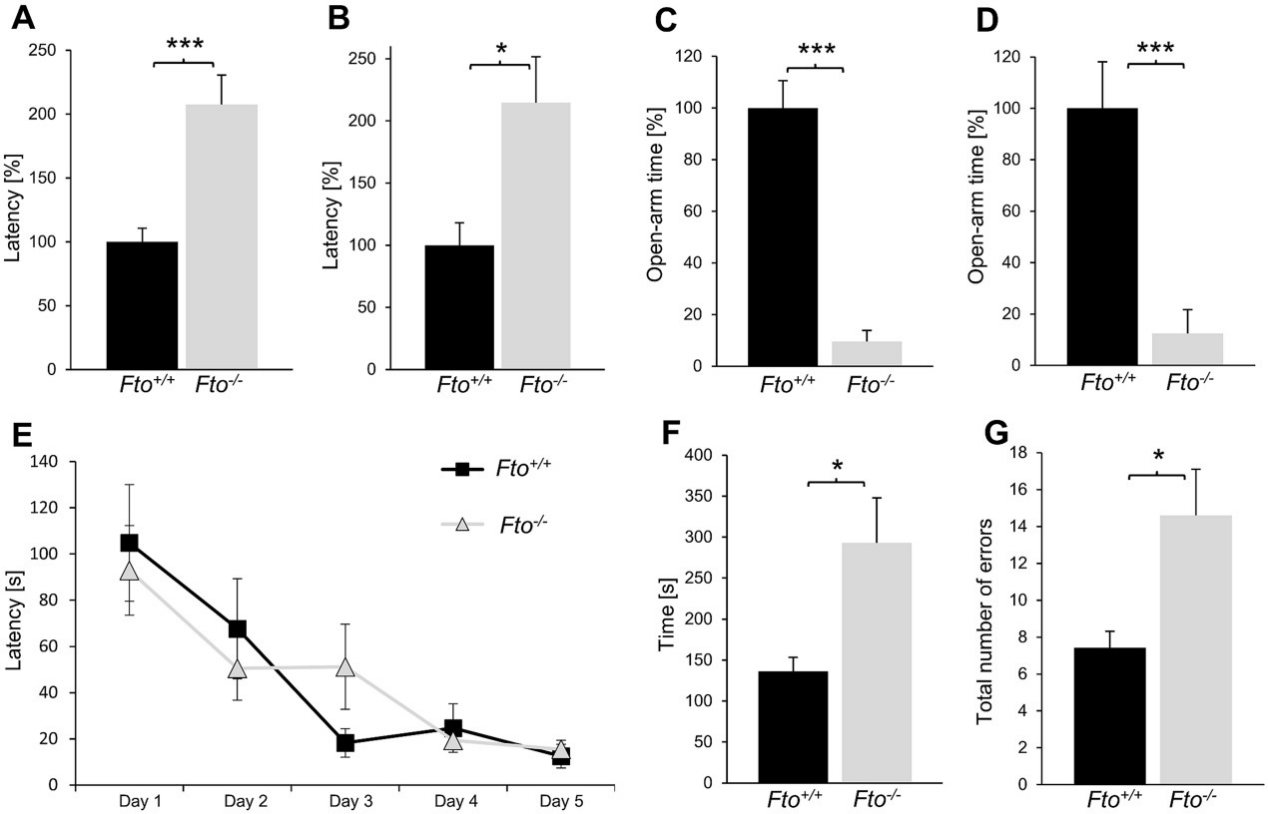
The loss of FTO leads to anxiety-like behavior and impairments in working memory. (A-B) The escape latency of 8 weeks old (A) and 16 weeks old (B) Fto^+/+^ and Fto^-/-^ mice in the cage emergence test indicated a higher anxiety level in Fto^-/-^ mice, n = 24 Fto^+/+^ / 20 Fto^-/-^ (A); n = 11 Fto^+/+^ / 18 Fto^-/-^ (B). (C-D) Measurements of the open arm time in the elevated plus maze test for 8 weeks old (C) and 16 weeks old (D) Fto^+/+^ and Fto^-/-^ mice revealed a higher anxiety level in Fto^-/-^ mice, n = 6 (C); n = 12 Fto^+/+^ / 9 Fto^-/-^ (D). (E) Fto^-/-^ mice show an unaltered escape latency during 5 days’ navigation training in the Morris water maze, n = 9 Fto^+/+^ / 12 Fto^-/-^. (F) Fto^-/-^ mice need more time to visit every single arm at least once in the radial eight-arm maze, n = 7 Fto^+/+^ / 5 Fto^-/-^. (G) The total number of memory errors in the radial eight-arm maze was higher for Fto^-/-^ mice, n = 7 Fto^+/+^ / 5 Fto^-/-^. Data are presented as mean ± S.E.M., unpaired t-test, *P < 0.05; **P < 0.01; ***P < 0.001.

A higher sensitivity to anxiogenic-like stimuli and increased anxiety-like behavior in mice are often associated with increased HPA axis activity. To establish whether the observed high corticosterone levels in Fto^-/-^ mice are linked to anxiety related behavior or an abnormal anxiety response, we tested the mice in two well-defined unconditional behavioral settings. In order to rule out that age-specific differences are present, which were described for behavioral tests in mice [22], different mouse groups were examined both at the age of 8 weeks, and at the age of 16 weeks. As demonstrated in Fig 2A and 2B, Fto deficiency significantly increased the time until the tested mice start to explore the larger barren cage in the cage emergence test. No age specific differences could be seen during this test. At an age of 8 weeks (Fig 2A) as well as at an age of 16 weeks (Fig 2B), Fto^-/-^ mice showed a more than two-fold higher latency time than Fto^+/+^ mice, indicating a higher anxiety level and a lower urge to explore new spaces in Fto^-/-^ mice.

To confirm this conspicuity, we further analyzed the anxiety levels of Fto^+/+^ and Fto^-/-^ mice in the elevated plus maze. The results demonstrated that Fto^-/-^ mice spent significant less time in the open arms than Fto^+/+^ mice independent of their age (Fig 2C and 2D). Furthermore, they spent significant more time in the closed arms indicating anxiety and confirming the results from the cage emergence test (S1A and S1B Fig). Taken together, these results demonstrate that Fto^-/-^ mice are more sensitive to anxiogenic-like stimuli.

Both increased corticosterone levels as well as phenotypic abnormalities, such as increased anxiety, are associated with impaired cognitive functions. Thus, we explored whether the increased anxiety caused by the loss of FTO could alter long-term memory in the Morris water maze and working memory in the eight-arm radial maze tests. The Fto^-/-^ mice displayed a similar escape latency during a 5-day training period in the Morris water maze, indicating no impairments in long-term memory (Fig 2E). However, testing working memory in the eight-arm radial maze revealed a higher number of errors (Fig 2G) and a longer period of time needed for visiting every single arm at least once (Fig 2F) in Fto^-/-^ mice compared to Fto^+/+^ mice. Together, these results suggest that Fto deficiency impairs working memory but not long-term memory in mice.

### The loss of FTO leads to impairment of neuronal differentiation in the hippocampus

**Fig 3:**
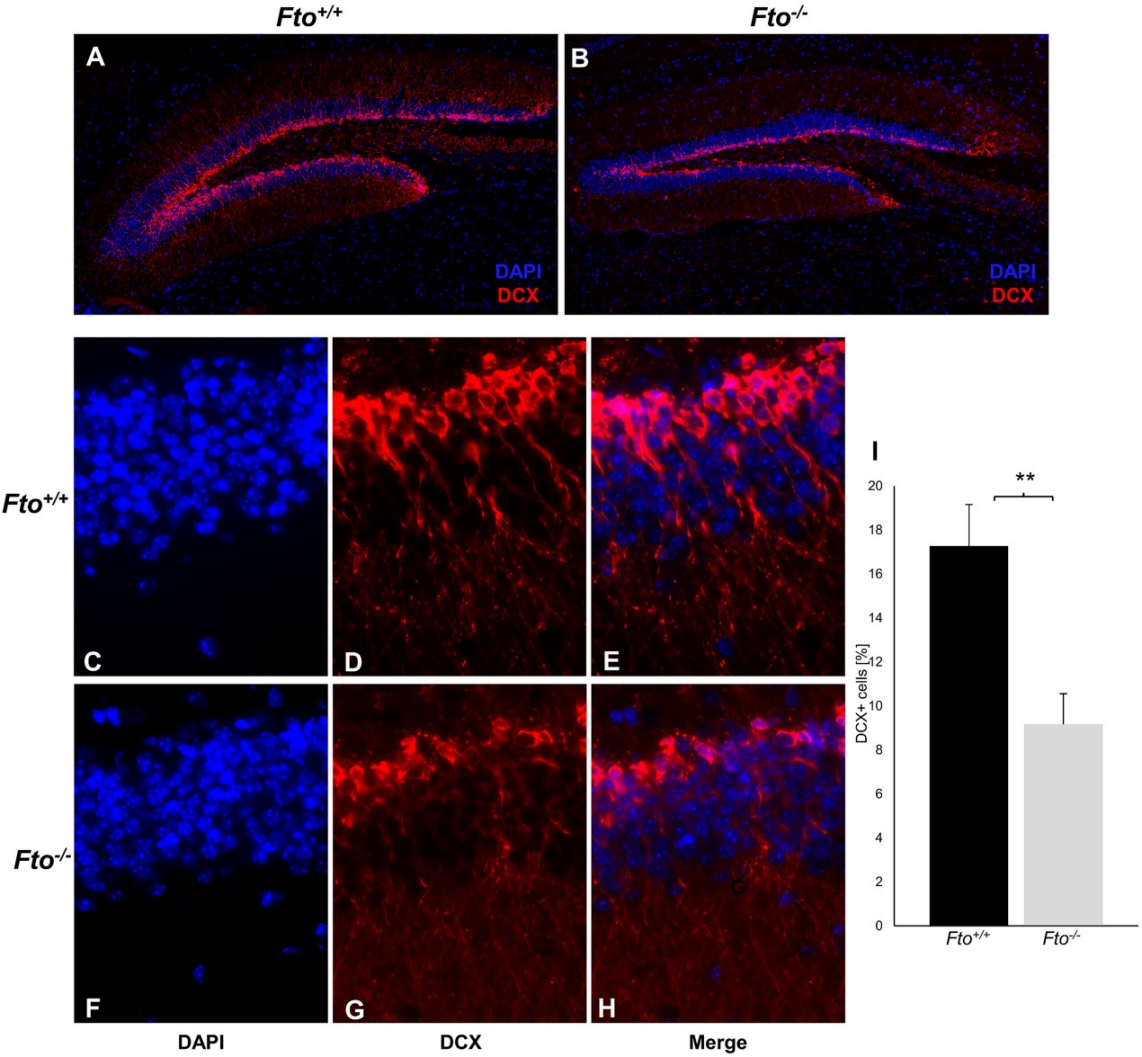
The loss of FTO leads to impairment of neuronal differentiation in the dentate gyrus of the hippocampus. (A-B) Representative overview images of whole dentate gyrus of the hippocampus of Fto^+/+^ and Fto^-/-^ mice.(C-H) Detailed representative immunofluorescence staining images show DCX^+^ neurons of the dentate gyrus in the hippocampus of Fto^+/+^ and Fto^-/-^ mice. (I) Quantitative analysis indicated that Fto^-/-^ mice had a lower percentage of DCX^+^ neurons per total cell number in the dentate gyrus than Fto^+/+^ mice, n = 4 Fto^+/+^ / 5 Fto^-/-^. Data are presented as mean ± S.E.M., unpaired t-test, **P < 0.01.

Both, hyperactivity of the HPA axis, as well as limitations of cognitive functions, are closely associated with changes in the hippocampus. In order to investigate whether the above described abnormalities in Fto^-/-^ mice are associated with changes in the hippocampus, the differentiation of cells in the subgranular zone was more closely examined. Therefore, doublecortin-positive cells were analyzed in the dentate gyrus of the hippocampus by immunofluorescence staining (Fig 3A–3H). Quantification revealed a significant lower number of doublecortin-positive cells in the subgranular zone of Fto^-/-^ mice (Fig 3I). This result suggests that the loss of FTO could lead to an impairment in the immature neuronal differentiation in the subgranular zone of the hippocampus.

### The loss of FTO leads to an altered ratio between proBDNF and mBDNF in the hippocampus and a breakdown of MAPK-pathway activity

**Fig 4:**
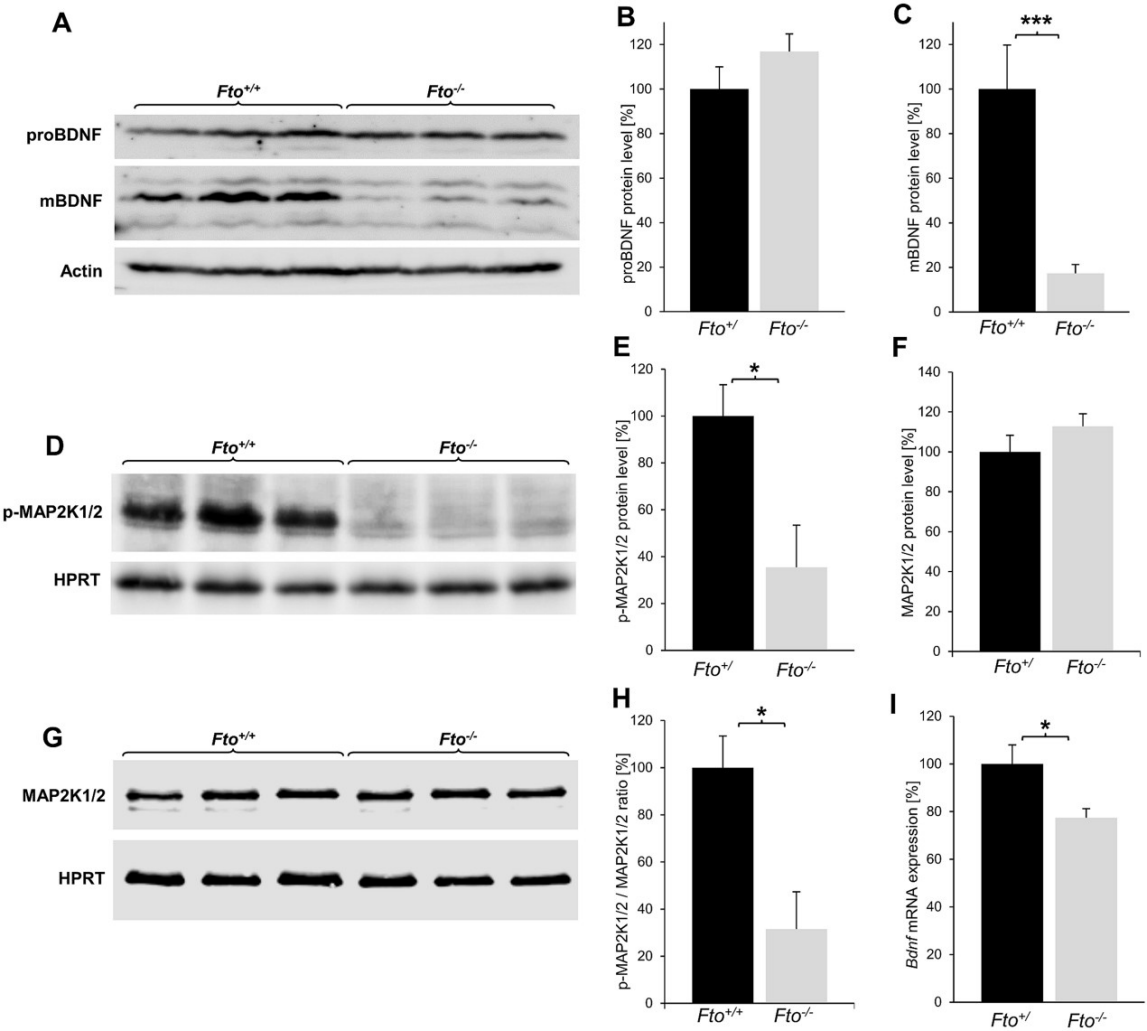
The loss of FTO leads to a misbalance of mBDNF to proBDNF in the hippocampus and a decreased downstream pathway activation. (A) Representative western blot images showing the protein levels of proBDNF and mBDNF with actin as loading control on one gel. For better clarity, the distance between the bands was cut out. (B-C) Quantification of images indicated significant lower mBDNF amounts and equal proBDNF amounts in the hippocampus of Fto^-/-^ mice, n = 6. (D) Representative western blot images of p-MAP2K1/2 with HPRT as loading control. (E) Quantification of images indicated a significant reduced MAPK pathway activity by revealing a decreased p-MAP2K1/2protein amount in the hippocampus of Fto^-/-^ mice, n = 4. (F-G) Western blot analysis of the nonphosphorylated form of MAP2K1/2 showed no change in Fto^-/-^ mice, n = 4. (H) Comparison of the nonphosphorylated and phosphorylated MAP2K1/2 amounts revealed a significant decreased of p-MAP2K1/2 to MAP2K1/2 ratio in the hippocampus of Fto^-/-^ mice. (I) Relative quantification results from real-time PCR analysis for Bdnf mRNA transcripts revealed a slight downregulation in Fto^-/-^ mice in the hippocampus, n = 8 Fto^+/+^ / 11 Fto^+/+^. Data are presented as mean ± S.E.M., unpaired t-test, *P < 0.05; ***P < 0.001.

All analyses carried out so far in this work are directly related to hippocampal functions: elevated ACTH and corticosterone values, anxiety disorder, as well as partially restricted cognitive abilities. BDNF is highly expressed in the hippocampus and serves essential functions in the mature brain in neuronal plasticity and is crucial for learning and memory processes [23, 24]. Thus, alterations in BDNF levels and signaling have been implicated in many neurological diseases [23]. To study, which molecular consequences Fto deficiency has on the hippocampus, we investigated the expression of RNA and the protein amount of mature BDNF and its precursor protein proBDNF by western blot analyses (Fig 4A). Real-time qPCR of hippocampal RNA revealed a slight but significant decrease in Bdnf mRNA within the hippocampus of FTO deficient mice (Fig 4I). However, the protein amount of the proBDNF isoform, which exerts proapoptotic activity as a specific ligand for the NGFR receptor, is not altered in the hippocampus of FTO deficient mice (Fig 4B). Interestingly, the analysis revealed that the amount of the posttranslational processed mature isoform of BDNF (mBDNF), which is an important positive regulator of cell survival and plasticity in the hippocampus, is significantly reduced in FTO deficient mice (Fig 4C).

In order to investigate whether the lower mBDNF amounts in the hippocampus of Fto^-/-^ mice leads to molecular consequences in the hippocampus, one of the most important signaling pathways, which is activated by mBDNF in the hippocampus, has been investigated. The mitogen-activated protein kinase (MAPK) signaling pathway depends on the activation of the mBDNF receptor NTRK2 and regulates various transcription factors responsible for growth, differentiation and function of neurons [25–27]. Western Blot analyses revealed a significantly lower amount of the phosphorylated and thus activated form of MAP2K1/2 in the hippocampus of Fto^-/-^ mice compared to Fto^+/+^ mice (Fig 4D). The relative amount of p-MAP2K1/2 in Fto^-/-^ mice is only about 35% compared to the amount in Fto^+/+^ mice (Fig 4E). Western blot analysis of the nonphosphorylated form of MAP2K1/2 showed no differences in Fto^-/-^ mice (Fig 4F–4G). Comparison of the nonphosphorylated and phosphorylated MAP2K1/2 amounts revealed a significant decreased p-MAP2K1/2 to MAP2K1/2 ratio in the hippocampus of Fto^-/-^ mice (Fig 4H). This result clearly demonstrates that the MAPK signaling pathway in the hippocampus of Fto^-/-^ mice has a lower activity than in Fto^+/+^ mice. This confirms that the amount of mBDNF is reduced in the hippocampus of Fto^-/-^ mice.

### FTO affects the processing of BDNF via MMP-9

**Fig 5:**
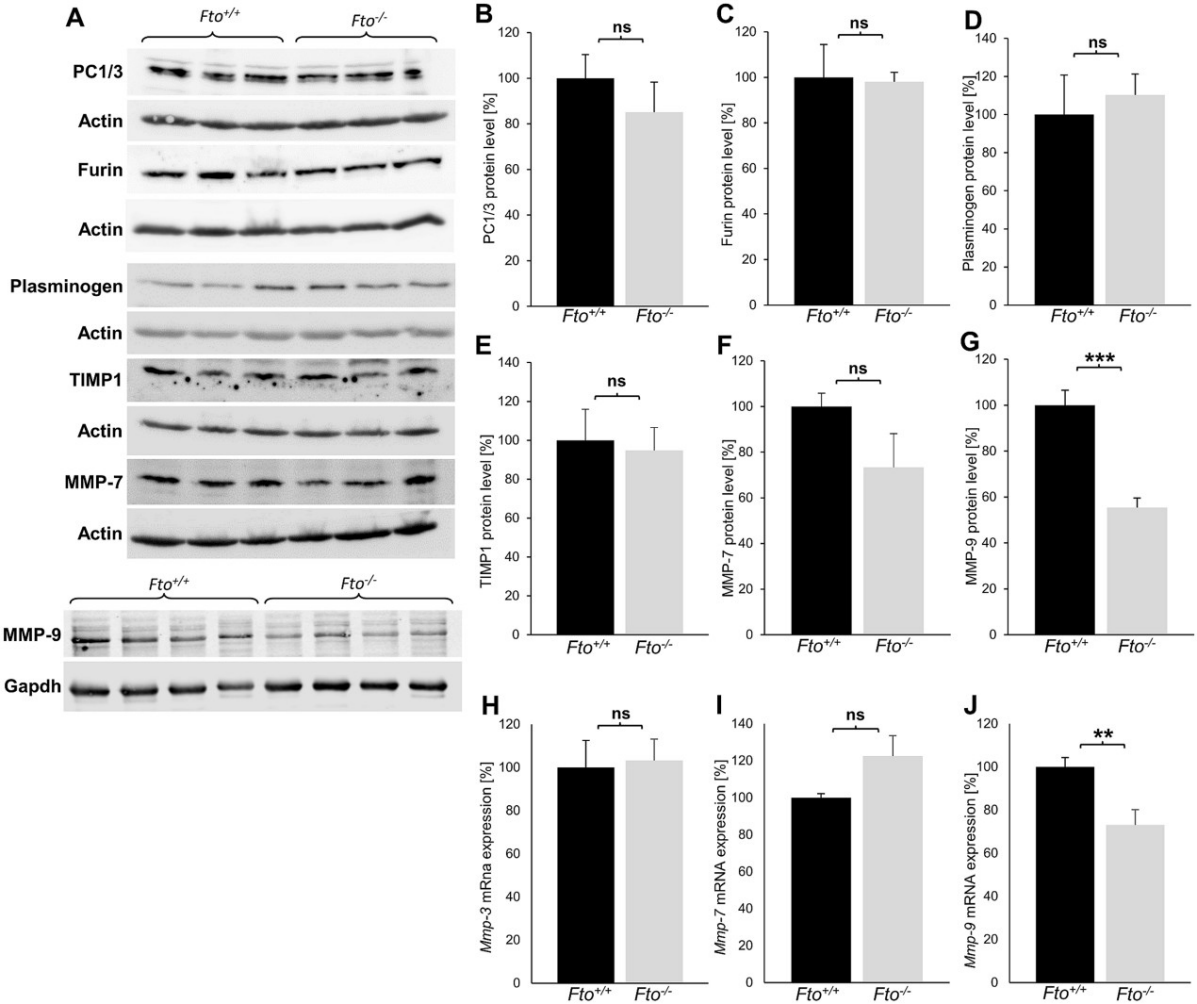
FTO seems to regulate BDNF processing through modulation of the MMP-9 amounts. (A) Representative western blot images for proteins involved in the cleavage of BDNF showing the protein levels of PC1/3, Furin, Plasminogen, TIMP1, MMP-7 and MMP-9 in the hippocampus. Actin or GAPDH were used as loading controls. (B-G) Quantification of western blots indicating no differences in the protein amounts of PC1/3 (B), Furin (C), Plasminogen (D), TIMP1 (E) and MMP-7 (F) between genotypes (n = 3) but a decreased protein amount of MMP-9 (G) in Fto^-/-^ mice (n = 7). (H-J) Quantification of Mmp-3 (H), Mmp-7 (I) and Mmp-9 (J) mRNA transcripts detected in the hippocampus by real-time PCR. The mRNA expression of Mmp-9 is reduced in the hippocampus of Fto^-/-^ mice, n = 6 Fto^+/+^ / 7 Fto^-/-^. Data are presented as mean ± S.E.M., unpaired t-test, **P < 0.01 ***P < 0.001.

The lower amount of mBDNF in the hippocampus of Fto^-/-^ mice and the unchanged proBDNF level indicate that FTO has an influence on the processing of BDNF. As with many neurotrophins, processing of BDNF is performed by different proteases and convertases. Therefore, the protein amounts of the most important proteases and convertases involved in the processing of BDNF in the hippocampus was quantified by western blot analyses in Fto^+/+^ and Fto^-/-^ mice (Fig 5A). These results revealed that the convertases PC1/3 and Furin show no differences in the amount of protein in the hippocampus of Fto^+/+^ and Fto^-/-^ mice (Fig 5B and 5C). Analyses for the proteins involved in extracellular BDNF processing Plasminogen, tissue-inhibitor of metalloproteinase 1 (TIMP1) and MMP-7 also showed no significant differences in their protein levels in the hippocampus of both genotypes (Fig 5D–5F). Interestingly, the amount of MMP-9 was significantly reduced in the hippocampus of Fto^-/-^ mice (Fig 5G). The MMP-9 amount in Fto^-/-^ mice is only about 55% of the normal amount in Fto^+/+^ mice. Thus, MMP-9 seems to be responsible for the lower proBDNF to mBDNF processing in the hippocampus of Fto^-/-^ mice. To further investigate whether FTO influences RNA levels of Mmps we performed a quantitative real-time PCR for the three most important Mmps: Mmp-3, Mmp-7 and Mmp-9. Expression analyses revealed that the RNA amounts of two matrix metalloproteinases Mmp-3 and Mmp-7 were not altered (Fig 5H and 5I). However, RNA of Mmp-9 was significantly reduced in expression in the hippocampus of Fto^-/-^ mice compared to Fto^+/+^ mice (Fig 5J). In summary, these results show that there is significantly less MMP-9 in the hippocampus of Fto^-/-^ mice, both at the RNA level and at the protein level.

## Discussion

This study demonstrates that the loss of FTO affects the function of the hippocampus due to an altered mBDNF/proBDNF ratio. Previous studies already showed that FTO plays an important role in the dopaminergic system in the midbrain, and that FTO deficiency impairs the dopamine receptor-dependent control of neuronal activity and behavioral responses [28]. Moreover, recent studies suggested that FTO influences transcripts involved in synaptic activity and memory-related processes [28, 29] and that the loss of FTO leads to reduced neurogenesis in the hippocampus [9]. All these studies strongly suggested that FTO plays important roles in modulating brain functions. In this work, we demonstrate that FTO is necessary for the correct function of the hippocampus by regulating BDNF processing.

Several groups showed that FTO controls growth and body weight and that FTO variants were associated with reduced brain volume and brain activity [2, 9, 30, 31]. Our investigations revealed that FTO influences corticosterone levels which might contribute to these abnormalities. In this context, it was described that chronic stress and high corticosterone levels in rodents results in reduced growth and lower birth weight [32, 33]. Nevertheless, long-lasting chronic high levels of corticosterone lead to fat accumulation, hyperglycemia and insulin resistance. However, this could not be demonstrated for Fto^-/-^ mice, suggesting that the HPA axis is not chronically activated, but rather shows a temporary overreaction to external stimuli. Thus, FTO seems to influence the sensitivity of the HPA axis rather than the function of the axis in general. Furthermore, it has been shown that chronically elevated corticosterone levels lead to reduced neuronal plasticity in the brain and impair glucose utilization making neurons more susceptible to metabolic damage [34–37]. Thus, Fto contributes to the correct functioning of the central nervous system by supporting the correct sensitivity of the neuroendocrine stress response.

A recent study reported that FTO governs hippocampal neurogenesis and cell differentiation [9]. In our work, the differentiation of cells in the hippocampal dentate gyrus was also analyzed, as it was shown that corticosterone influences the differentiation of precursor cells in the hippocampus [38]. We show that the loss of FTO leads to a lower number of differentiating precursor cells in the hippocampus. This differentiation defect could be caused by both the increased corticosterone levels, as well as by the disturbed processing from proBdnf to mBdnf due to the loss of FTO. Furthermore, this structural damage of the hippocampus is usually associated with reduced cognitive functions, as well as mental illnesses. In our study, we demonstrate that the loss of FTO leads to increased anxiety and impairments in working memory in mice. However long-term memory seems to be not affected by Fto, which could be due to the fact that for the long-term memory different brain areas are responsible, whereas for the working memory above all the hippocampus is of crucial importance [39, 40]. Several groups have shown that this mental disorder is often associated with a reduced number of doublecortin-positive cells in the hippocampus indicating a restricted differentiation of the neurons [41, 42]. Accordingly, the increased anxiety in Fto^-/-^ mice could be caused by the restricted neuronal differentiation in the hippocampus.

All the functions limited by the loss of FTO are attributed to the tasks of the hippocampus. As a cause for these impairments, we identified a processing defect of the neurotrophin BDNF. Previous studies suggested that FTO could influence several components of the BDNF pathway, however the isoforms of BDNF were not investigated [9, 43]. Here we demonstrate that loss of FTO leads to a reduced amount of MMP-9 in the hippocampus, resulting in a disturbed ratio of proBDNF and mBDNF. While the amount of the proBDNF isoform is not altered on the protein level despite a slightly reduced expression of Bdnf on mRNA level, the amount of processed mBDNF isoform is significantly reduced in Fto mice. This leads to a reduced activation of the MAPK signaling pathway, which is responsible for the differentiation, growth, cell invasion and protection of neurons. The mechanism by which FTO regulates the amount of MMP-9 cannot be clearly determined by our analyses. However, it has been shown that FTO is able to regulate MMPs to promote cell migration in the endometrial cancer cell line Ishikawa. In this study a knockdown of FTO by siRNA resulted in a decreased MMP9 amount and thus reduced cell migration in endometrial cells, indicating regulatory role of FTO in MMP production [44]. Regarding the function of FTO as a demethylase, it could only be shown at the DNA level that epigenetic modifications influence the expression of MMP-9. Importantly, the treatment of a lymphoma cell line with a DNA methylation inhibitor resulted in reduced methylation of the MMP-9 promoter, resulting in increased expression of MMP-9 [45]. For both humans and the mouse, various possible RNA methylation sites on the MMP-9 mRNA could be predicted to date. While m6A methylation is suspected to take place on each of the 13 Mmp-9 exons in mice, m6A sites in humans were only predicted on the exons 9, 10 and 11 [46]. Although there is no functional analysis, that directly shows that Fto has an impact on m6A in MMP9 transcripts, the fact that methylation sites are present on the MMP9 transcript, make it very likely that Fto regulates the expression of MMP9 via the demethylation of these site We hypothesize that FTO controls the amount of MMP-9 via its function as RNA demethylase in the hippocampus. Considering that FTO ensures proper hippocampus function by regulating BDNF processing via governing the hippocampal MMP-9 amount, we propose FTO as a possible new target to develop novel approaches for the treatment of diseases associated with hippocampal disorders via regulating the processing of BDNF. In parallel, we also would like to make the point that any anti-obesity therapy via blocking FTO function can have negative effects on the proper function of the hippocampus.

## Materials and methods

### Ethics statement

All animal experiments were performed in accordance with the relevant national guidelines for the Care and Use of Laboratory Animals (LANUV) and with approval from the authority for animal work at Heinrich-Heine-University Düsseldorf, Germany (Permit number 84–02.04.2011.A391).

### Animal care

Fto mutant mice are described [2]. Analyses were performed with mice of breeding pairs of Fto^+/-^ C57BL/6J mice crossed with Fto^+/-^ NMRI mice. Because Fto^+/—^mice has a wildtypic phenotype, only Fto^+/+^ and Fto^-/-^ mice where compared [2]. Only male mice were used for the analysis to prevent hormonal fluctuation that occurs during the estrous cycle and could potentially affect animal behavior [47]. Mice were housed at 22–24 °C on a 12/12 h dark-light cycle with food and water ad libitum. At the age of 16 weeks animals were killed by cervical dislocation, organs and blood were isolated and collected. Organs needed for further experiments were stored or handled according to the method.

### Antibodies

We used the following primary antibodies: rabbit anti-mBdnf and proBdnf (Santa Cruz Biotechnology, sc-546), rabbit anti phospho-MEK1/2 (Ser217/221) (Cell Signaling Technology, #9121), rabbit anti MEK1/2 (Cell Signaling Technology, #9122), rabbit anti-Actin (A2066; Sigma-Aldrich), rabbit anti-Hprt (ab10479; Abcam), rabbit anti-Gapdh (Cell Signaling; 2118), HRP anti-rabbit (P0448; Dako), mouse anti-Mmp7 (Santa Cruz; sc-515703), mouse anti-Mmp9 (Santa Cruz; sc-393859), mouse anti-Mmp3 (Santa Cruz; sc-21732), mouse anti-Furin (Santa Cruz; sc-133142), mouse anti-Plasminogen (Santa Cruz; sc-376324), mouse anti-Timp1 (Santa Cruz; sc-365905), rabbit anti-PC1/3 (Cell Signaling; 11914), IRDye 800CW anti-mouse (LI-COR; 925–32210) and IRDye 680RD anti-rabbit (LI-COR; 925–68071)

### Elevated plus maze (EPM)

The elevated plus maze is the most commonly used rodent model of anxiety [48]. It is based on rodents’ natural aversion of open spaces and leads to a behavior, which involves avoidance of open areas by confining movements to enclosed spaces. Our mouse elevated plus mazes was situated in a separate brightly lit room that was illuminated with overhead lights, which provided a light density of approximately 300 lx at the center and the arms of the EPM. The EPM consisted of a cross-shaped platform (height: 60 cm) with four arms (width: 6 cm; length: 40 cm), two of which were enclosed by walls 30 cm in height. The mice from each group were placed in the center of this cross facing an open arm and were free to explore the EPM for 5 minutes and their activity were videotaped. Anxiety was indicated by an increase in the proportion of time spent in the closed arms, and an increase in the proportion of entries into the closed arms.

### Cage emergence test

The cage emergence test was performed as previously described [49]. In short, the apparatus consisted of a Macrolon type I cage (204 cm^2^) with a hole (∅ 4 cm) in one of the opaque side walls. There was no lid on the cage. A mouse was placed inside the cage with its back to the opening and the time to escape from the cage (all four feet outside the cage) into a bright and open space (300 lx) was registered.

### Morris water maze test

The Morris water maze was performed as previously described [50]. A round basin with a diameter of approx. 130 cm was used for carrying out the test in a separate brightly lit room that was illuminated with overhead lights, which provided a light density of approximately 300 lx at the water surface. It was filled with 27 °C warm water up to a height of 25 cm. The water was made opaque by adding non-toxic white titanium dioxide. A hidden white platform with a diameter of 10 cm was placed 1 cm under the water surface. For orientation, four different visual orientation points were placed crosswise at the edge of the basin. Furthermore, the animals were able to orient themselves at distal points of orientation in the room consisting of shelves, table, door and wall. The test took place over a period of 5 days, each animal being tested once a day. It was put into the water daily at different starting points with the head to the edge of the basin and could explore the basin for a maximum of 180 seconds. If after this time the platform could not be reached, the animal was led manually to the platform. After reaching the platform the animals were allowed to linger on this for 10 seconds to orient themselves and to remember the position. The entire experiment was video-documented with the aid of a camera mounted above the basin and then evaluated by comparing the time until the platform was reached and compared between the animals.

### Eight-arm radial maze test

The eight-arm redial maze test was performed as previously described [51]. It consisted of an octagonal central platform and eight outstretched arms. The individual arms had an angle of 45 ° to one another and were surrounded by opaque side walls. In order to ensure the orientation of the animals in the radial maze, four visual landmarks were placed crosswise at the ends of four of the eight arms. In addition, further distal visual orientation points such as shelves, table, door and wall were located in the experimental room. The movements of the animals during the experiment were documented by a video camera mounted about 1.5 meters above the radial maze. At the beginning of the experiment, the mice were individually placed in the center of the maze, which they were allowed to explore freely for a maximum of 5 minutes. As a matter of fact, the mice would enter each arm from the center in the exploration of the maze, before they would re-enter an already explored arm. Entering an already explored arm before all 8 arms were explored once was defined as an error. In addition, the time required by the mouse to find at least once all eight arms was measured. Both an increased number of re-entries in already visited arms as well as an increased expenditure of time for the exploration of each arm were understood as a deficit in the performance of the working memory. The evaluation was made after the tests on the basis of the video recordings. The radial maze was cleaned with 70% ethanol between the individual test runs.

### Measurement of corticosterone and ACTH

Blood was obtained between 9 am and 10 am from 16 weeks old mice. Mice were rapidly anaesthetized by being placed in a chamber containing isoflurane in oxygen. As soon as anesthesia was achieved, blood was quickly obtained via cardiac puncture. Plasma from each blood sample was obtain by centrifugation at 2000 g for 10 minutes, and stored at -80 °C until assay was performed. The corticosterone and ACTH level in plasma was detected using a commercial enzyme-linked immunosorbent assay (ELISA) kit (Abnova, Taipei, Taiwan), according to manufacturer’s instructions.

### Preparation of the hippocampus

Animals were killed and brains were immediately removed. The hippocampus was dissected out and snap-frozen in liquid nitrogen and stored at −80 °C for further analyses.

### Protein extraction

Tissues were thawed and homogenized in radioimmunoprecipitation buffer (150 mM sodium chloride, 50 mM Tris-HCl, pH 7.4, 0.1% sodium deoxycholate, and 1 mM EDTA) containing PhosSTOP phosphatase inhibitor (Roche #04906845001) and complete Mini protease inhibitor (Roche #11836153001) using a Precellys 24 high-throughput homogenizer (Bertin Technologies, Rockville, Washington D.C., USA). Protein content was measured by the Bradford method, and samples were normalized. 20 mg of total protein was separated by SDS-PAGE on polyacrylamide gels (15%) and transferred to a polyvinylidene fluoride membrane (Bio-Rad Laboratories, Inc.). The membrane was probed with primary antibodies listed in the antibody section. Anti-Actin, anti-Gapdh and anti-Hprt antibodies were used as loading controls. Proteins were detected with secondary antibodies conjugated to horseradish peroxidase (RPN4201 and RPN4301) and the ECL detection kit (both GE Healthcare) or by near-infrared fluorescent dyes (LI-COR). Visualization of protein bands was realized by LAS-4000 mini (Fujifilm) or with an Odyssey near-infrared scanner (Licor Biosciences). Band intensities were measured by using ImageJ (National Institutes of Health).

### Real-time PCR Analysis

Hippocampi tissues were thawed and RNA was isolated using RNeasy Kit (Qiagen #74104) and RNase-Free DNase Set (Qiagen # 79254). Isolated RNA was converted into cDNA by using Expand Reverse Transcriptase (Roche # 11785826001). Quantitative real-time PCR was carried out by employing a Step One Real-Time PCR System Thermal Cycling Block (Applied Biosystems #4376357) and the TaqMan Universal PCR Master Mix, No AmpErase UNG (Applied Biosystems #4324020). The following primer/TaqMan probe sets were used: Gapdh (Assay ID: Mm99999915_g1), Bdnf (Assay. ID: Mm04230607_s1). Real-time PCR was carried out with 50 ng of hippocampal cDNA of each sample in triplicate reactions in a 20 μl volume containing 100 nM primers and 50 nM probe. Cycling conditions were 50°C for 2 minutes and 95°C for 10 minutes, followed by a 40-cycle amplification of 95°C for 15 seconds and 60°C for 1 minute. The analysis of real-time data was performed by using included StepOne Software version 2.0.

### Figure preparation

Figure preparation was performed by using Photoshop CS2 (Adobe) and collages of the all images for figure preparation were arranged using Illustrator CS2 (Adobe) and PowerPoint 2016 (Microsoft).

### Statistical data

Data are presented as means ± SEM. Two-tailed Student’s t test was performed for all compared data by using Excel (Microsoft). A p-value < 0.05 was considered to be statistically significant (one asterisk), a p-value < 0.01 was regarded as statistically very significant (two asterisks), and a p-value < 0.001 was considered to be highly significant statistically (three asterisks).

## Supporting information

S1 Fig The loss of Fto leads to anxiety-like behavior. (A-B) Measurements of the closed-arm time in the elevated plus maze test for 8 weeks old (A) and 16 weeks old (B) Fto^+/+^ and Fto^-/-^ mice revealed a higher anxiety level in Fto^-/-^ mice, n = 6 (A); n = 12 Fto^+/+^ / 9 Fto^-/-^.
(PDF)
