# Supplementary material for: FTO affects hippocampal function by regulation of BDNF processing
Source: PLoS One. 2019 Feb 7;14(2):e0211937. doi: 10.1371/journal.pone.0211937 (PMC6366932; doi:10.1371/journal.pone.0211937)
Supplement: S1 Fig — (A-B) Measurements of the closed-arm time in the elevated plus maze test for 8 weeks old (A) and 16 weeks old (B) Fto+/+ and Fto-/- mice revealed a higher anxiety level in Fto-/- mice, n = 6 (A); n = 12 Fto+/+ / 9 Fto-/-. (PDF) [file pone.0211937.s001.pdf]

Supplement Figure 1

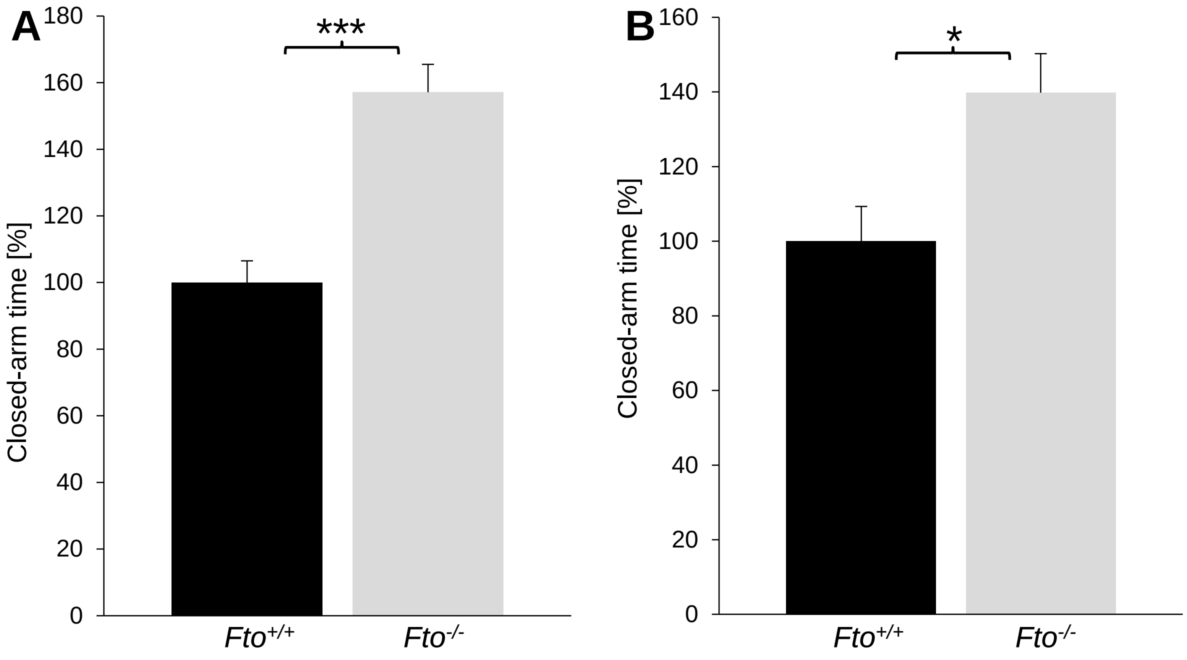

S1 Fig. The loss of Fto leads to anxiety-like behavior. (A-B) Measurements of the closed-arm time in the elevated plus maze test for 8 weeks old (A) and 16 weeks old (B) $Fto^{+/+}$ and $Fto^{-/-}$ mice revealed a higher anxiety level in $Fto^{-/-}$ mice, (A) n = 6 ; (B) n = 12 $Fto^{+/+}$ / 9 $Fto^{-/-}$.
